# Genome Sequence of Clostridium acetobutylicum GXAS18-1, a Novel Biobutanol Production Strain

**DOI:** 10.1128/genomeA.00033-15

**Published:** 2015-03-05

**Authors:** Xinchun Mo, Jianxin Pei, Yuan Guo, Lihua Lin, Lixin Peng, Chan Kou, Danmin Fan, Hao Pang

**Affiliations:** a National Engineering Research Center for Non-Food Biorefinery, Guangxi Academy of Science, Nanning, Guangxi, China; b Department of Life Science, Lijiang Teacher’s College, Lijiang, Yunnan, China

## Abstract

Clostridium acetobutylicum is an organism involved in the production of acetone and butanol by traditional acetone-butanol-ethanol fermentation (ABE). We report the draft genome sequence of C. acetobutylicum strain GXAS18-1, which can produce ABE directly from cassava flour*.*

## GENOME ANNOUNCEMENT

Clostridia, well-known commercial biobutanol production organisms, have the capability to convert a wide variety of renewable biomass and agricultural waste materials to butanol and ethanol. Clostridium acetobutylicum is one group of *Clostridium* strains that is generally used to produce acetone and butanol through traditional acetone-butanol-ethanol fermentation (ABE) (1). Previous studies have focused on the isolation of these organisms and their adoption for various feedstock and fermentation processing functions (2, 3). However, the industrial application of Clostridium acetobutylicum strains for high-performance solvent yields and their extreme tolerance for butanol still require more investigation. *C.* a*cetobutylicum* GXAS18-1 is a special butanol fermentation strain that can produce ABE directly from cassava flour by the addition of ammonium acetate. Here, we briefly describe the draft genome sequence of C. acetobutylicum GXAS18-1.

The genomic DNA from C. acetobutylicum GXAS18-1 (deposited in the China Center for Type Culture Collection [CCTCC] [accession number CCTCC M2011280]) was sequenced using an Illumina MiSeq platform (Illumina, San Diego, CA) with a paired-end library. The SOAPaligner 2.20 and SOAPdenovo 1.3 software (BGI bio, Shenzhen, China) were used to perform quality trimming and *de novo* assembly of the reads, respectively. The resulting contigs were reordered using SOAPdenovo 1.3 and C. acetobutylicum ATCC 824 as a reference genome (GenBank accession number NC_003030.1).

Potential coding sequences (CDSs) were predicted using RAST (4), and the assignment of protein functions was performed by searching against the GenBank and Pfam (5) databases using BLASTp (6), while single-nucleotide polymorphisms (SNPs) were identified using MUMmer 3.06 (7). Ribosomal RNAs, tRNAs, and other RNAs were identified using BLASTn, tRNAscan-SE version 1.21 (8), and RNAmmer 1.2 (9), respectively. The orthologous genes between C. acetobutylicum GXAS18-1 and C. acetobutylicum ATCC 824 were identified using SEED functional comparison (10). The draft genome of C. acetobutylicum GXAS18-1 consists of 49 contigs ranging in size from 590 to 924,670 bases, resulting in a total genome size of 3,796,049 nucleotides, with average genome coverage of 100-fold and a G+C content of 30.79%. Whole-genome SNP scan revealed that 50,702 SNP loci, which were identified in the *C. acetobutylicum* GXAS18-1 genome. Among them, 43,248 SNPs were found in the gene region accounting for the entire SNP set of 85.30%, whereas 7,454 SNPs are in the intergenic region accounting for 14.70% of the entire SNP set. The chromosome of *C. acetobutylicum* GXAS18-1 contains 3,600 CDSs and 3,648 genes, like other *Clostridium*, 3 noncontiguous rRNAs (5S, 16S, and 23S rRNA), 41 tRNAs, and 48 other RNAs. The *C. acetobutylicum* GXAS18-1 chromosome exhibits a high level of synteny to C. acetobutylicum ATCC 824, with the exception of 104 unique genes in the former chromosome and 64 genes in the latter, respectively. Furthermore, 2,306 functional genes were identified by SEED functional comparison involved in the metabolic subsystem, in which most of them were involved in carbohydrate (335 genes), amino acid and derivative (277 genes), and cofactor (244 genes) metabolic pathways, followed by RNA metabolism (153 genes) and the cell wall and capsule (150 genes). Others were involved in life cycle metabolism. A significant difference between C. acetobutylicum ATCC 824 and GXAS18-1 is that a clustered regularly interspaced short palindromic repeats (CRISPR) system is present in the GXAS18-1 genome but not in the C. acetobutylicum ATCC 824 genome.

### Nucleotide sequence accession numbers.

This whole-genome shotgun project has been deposited at DDBJ/EMBL/GenBank under the accession numbers JRWL01000001 to JRWL01000049 (BioProject number PRJNA263987).
